# Retraction Note to: Expansion of monocytic myeloid-derived suppressor cells ameliorated intestinal inflammatory response by radiation through SOCS3 expression

**DOI:** 10.1038/s41419-022-05295-6

**Published:** 2022-09-29

**Authors:** You Yeon Choi, Ki Moon Seong, Hyun Jung Lee, Seung Sook Lee, Areumnuri Kim

**Affiliations:** 1 Laboratory of Biodosimetry, National Radiation Emergency Medical Center, KIRAMS, Seoul, 01812 Korea; 2 Laboratory of Radiation Exposure and Therapeutics, National Radiation Emergency Medical Center, KIRAMS, Seoul, 01812 Korea; 3 Department of Pathology, Korea Cancer Center Hospital, Korea Institute of Radiological & Medical Science, Seoul, 01812 Korea

**Keywords:** Stress signalling, Super-resolution microscopy

Retraction to: *Cell Death and Disease* 10.1038/s41419-021-04103-x, published online 03 September 2021

The authors have retracted this article. After publication the authors found that the staining done in Figure 5D had not been done with the antibody SOCS3 as stated in the article. The authors have, therefore, lost confidence in their results. All authors agree to this retraction.

